# Supplementary material for: Genetic disruption of WASHC4 drives endo-lysosomal dysfunction and cognitive-movement impairments in mice and humans
Source: eLife. 2021 Mar 22;10:e61590. doi: 10.7554/eLife.61590 (PMC7984842; doi:10.7554/eLife.61590)
Supplement: Transparent reporting form [file elife-61590-transrepform.pdf]

eLIFE
Westbrook Centre, Milton Road
Cambridge CB4 1YG
UK
P 01223 855340
W elifesciences.org
T @elife

# *eLife's* transparent reporting form

We encourage authors to provide detailed information *within their submission* to facilitate the interpretation and replication of experiments. Authors can upload supporting documentation to indicate the use of appropriate reporting guidelines for health-related research (see EQUATOR Network), life science research (see the BioSharing Information Resource), or the ARRIVE guidelines for reporting work involving animal research. Where applicable, authors should refer to any relevant reporting standards documents in this form.

If you have any questions, please consult our Journal Policies and/or contact us: editorial@elifesciences.org.

**Sample-size estimation**

- You should state whether an appropriate sample size was computed when the study was being designed
- You should state the statistical method of sample size computation and any required assumptions
- If no explicit power analysis was used, you should describe how you decided what sample (replicate) size (number) to use

Please outline where this information can be found within the submission (e.g., sections or figure legends), or explain why this information doesn't apply to your submission:

We did not pre-calculate sample size using a statistical framework, but based our sample sizes on previous published work that used the same or similar assays, such as Spence *et al*., 2019 (Nat Commun. 2019 Jan 23;10(1):386. doi: 10.1038/s41467-019-08288-w) and Geladaki *et al*., 2019 (Nat Commun. 2019 Jan 18;10(1):331. doi: 10.1038/s41467-018-08191-w) for proteomic analyses, Uezu *et al*., 2019 (Elife. 2019 Dec 12;8:e50712. doi: 10.7554/eLife.50712) for imaging analyses, and Kim *et al*., 2014 (Elife. 2014 Jul 8;3:e02839. doi: 10.7554/eLife.02839) for behavioral analyses.

**Replicates**

- You should report how often each experiment was performed
- You should include a definition of biological versus technical replication
- The data obtained should be provided and sufficient information should be provided to indicate the number of independent biological and/or technical replicates
- If you encountered any outliers, you should describe how these were handled
- Criteria for exclusion/inclusion of data should be clearly stated
- High-throughput sequence data should be uploaded before submission, with a private link for reviewers provided (these are available from both GEO and ArrayExpress)

Please outline where this information can be found within the submission (e.g., sections or figure legends), or explain why this information doesn't apply to your submission:

We provide replicate information in each figure legend, and in the Quantification and Statistical Analysis section of the Materials and Methods. Criteria for outlier identification and exclusion from the proteomic data sets are stated in the Materials and Methods sections.

**Statistical reporting**

- Statistical analysis methods should be described and justified
- Raw data should be presented in figures whenever informative to do so (typically when N per group is less than 10)
- For each experiment, you should identify the statistical tests used, exact values of N, definitions of center, methods of multiple test correction, and dispersion and precision measures (e.g., mean, median, SD, SEM, confidence intervals; and, for the major substantive results, a measure of effect size (e.g., Pearson's r, Cohen's d)
- Report exact p-values wherever possible alongside the summary statistics and 95% confidence intervals. These should be reported for all key questions and not only when the p-value is less than 0.05.

Please outline where this information can be found within the submission (e.g., sections or figure legends), or explain why this information doesn't apply to your submission:

We report full statistical analyses in each figure legend. Further information can be found in the Quantification and Statistical Analysis section of the Materials and Methods.

(For large datasets, or papers with a very large number of statistical tests, you may upload a single table file with tests, Ns, etc., with reference to sections in the manuscript.)

**Group allocation**

- Indicate how samples were allocated into experimental groups (in the case of clinical studies, please specify allocation to treatment method); if randomization was used, please also state if restricted randomization was applied
- Indicate if masking was used during group allocation, data collection and/or data analysis

Please outline where this information can be found within the submission (e.g., sections or figure legends), or explain why this information doesn't apply to your submission:

Group allocation can be found in the Quantification and Statistical Analysis section of the Materials and Methods. Masking for group allocation, data collection, and/or data analysis is stated in the description of each method in the Materials and Methods.

**Additional data files ("source data")**

- We encourage you to upload relevant additional data files, such as numerical data that are represented as a graph in a figure, or as a summary table
- Where provided, these should be in the most useful format, and they can be uploaded as "Source data" files linked to a main figure or table
- Include model definition files including the full list of parameters used
- Include code used for data analysis (e.g., R, MatLab)
- Avoid stating that data files are "available upon request"

Please indicate the figures or tables for which source data files have been provided:

Source data for Figure 1 WASH1-BioID proteomics can be found in "S1_WASH_BioID_Results.xlsx." Source data for Figure 2 Western blots can be found in "Source_Data_Blots_v2.pdf." Source data for Figures 2-3 TMT proteomics can be found in "S2_Swip_TMT_Protein_GLM_Results.xlsx," and "S3_Swip_TMT_Module_GLM_Results.xlsx." We have deposited these data, along with source code, to GitHub: https://github.com/twesleyb/SwipProteomics.
